# Association of Locomotor Activity During Sleep Deprivation Treatment With Response

**DOI:** 10.3389/fpsyt.2020.00688

**Published:** 2020-07-21

**Authors:** Jerome Clifford Foo, Lea Sirignano, Nina Trautmann, Jinhyuk Kim, Stephanie H. Witt, Fabian Streit, Josef Frank, Lea Zillich, Andreas Meyer-Lindenberg, Ulrich Ebner-Priemer, Claudia Schilling, Michael Schredl, Yoshiharu Yamamoto, Maria Gilles, Michael Deuschle, Marcella Rietschel

**Affiliations:** ^1^Department of Genetic Epidemiology in Psychiatry, Central Institute of Mental Health, Medical Faculty Mannheim, University of Heidelberg, Mannheim, Germany; ^2^Department of Psychiatry and Psychotherapy, Central Institute of Mental Health, Medical Faculty Mannheim, University of Heidelberg, Mannheim, Germany; ^3^Department of Child and Adolescent Psychiatry and Psychotherapy, Central Institute of Mental Health, Medical Faculty Mannheim, University of Heidelberg, Mannheim, Germany; ^4^Department of Informatics, Graduate School of Integrated Science and Technology, Shizuoka University, Shizuoka, Japan; ^5^Department of Sport and Sport Science, Karlsruhe Institute of Technology, Karlsruhe, Germany; ^6^Department of Physical and Health Education, Graduate School of Education, The University of Tokyo, Tokyo, Japan

**Keywords:** locomotor activity, sleep deprivation treatment, depression, mood, treatment response

## Abstract

Disrupted circadian rhythms and sleep patterns are frequently observed features of psychiatric disorders, and especially mood disorders. Sleep deprivation treatment (SD) exerts rapid but transient antidepressant effects in depressed patients and has gained recognition as a model to study quick-acting antidepressant effects. It is of interest how locomotor activity patterns during SD might be associated with and potentially predict treatment response. The present study is an analysis of locomotor activity data, previously collected over a 24 h period, to examine the night of SD (Trautmann et al. 2018) as mood disorder patients suffering from a depressive episode (n = 78; after exclusions n = 59) underwent SD. In this exploratory analysis, the associations between response to SD, locomotor activity, and subjective mood during the 24 h period of SD were explored. Higher levels of activity overall were observed in non-responders (n = 18); in particular, non-responders moved more during the evening of SD until midnight and remained high thereafter. In contrast, activity in responders (n = 41) decreased during the evening and increased in the morning. Subjective mood was not found to be associated with locomotor activity. The window of data available in this analysis being limited, additional data from before and after the intervention are required to fully characterize the results observed. The present results hint at the possible utility of locomotor activity as a predictor and early indicator of treatment response, and suggest that the relationship between SD and locomotor activity patterns should be further investigated.

## Introduction

Disruption of sleep and circadian rhythms are often reported as features of psychiatric disorders, and chronotherapeutic treatments targeting the circadian system have shown the ability to affect disease symptoms (1–5). Therapeutic sleep deprivation (SD), involving a night without sleep, is one such treatment which has been found to rapidly induce an antidepressant response in depressed patients. Interestingly, this effect is transient with depressive symptoms reappearing directly after recovery sleep (6–10). While response to pharmacological antidepressants often takes weeks to emerge, SD can be quickly administered in a controlled fashion, providing an efficient model to study fast-acting antidepressants. SD Response is thought to be associated with various factors; several hypotheses about mechanisms of action have been proposed (11–14), with a popular one suggesting that SD acts through resetting circadian processes which are dysregulated in depression (15–19).

Altered psychomotor behavior has been found to be associated with many psychiatric disorders and disrupted circadian rhythmicity observed in affective disorders can also be reflected in locomotor activity patterns (20–29). While there is evidence suggesting that better mood is correlated with increased (everyday/non-exercise) locomotor activity (30–34), it is becoming apparent that the relationship between locomotor activity with psychiatric disorders is complex. For example, a pedigree study examining genetic contributions to circadian rhythms segregating by bipolar disorder (BD) severity showed that BD individuals consistently demonstrated lower levels of activity than their non-BD relatives (35). Studies in unrelated patients showed that mania patients display more complex activity patterns in the active morning period compared to depressed patients (36). Meanwhile, compared to healthy controls, individuals suffering from depression display lower mean activity levels (27, 36, 37) but higher variability over the whole day as well as in the active morning period (36). The characterization of these relationships is becoming increasingly feasible thanks to recent developments in wearable and mobile sensing technologies, especially in easy and non-invasive monitoring of locomotor activity (38–40) using wristwatch-type actigraph, making it a promising method which can be implemented with high compliance in clinical contexts.

It is of interest to explore how locomotor activity of patients changes through SD and whether this change is associated with response. Various actigraphy studies have been carried out in depressed populations (21–23, 30, 40, 41) but only a few studies in the SD context have assessed aspects of locomotor activity during treatment, and those in limited samples (18, 42–45). One study found that several manic-depressive patients emerging from depressive phases advanced awakening times, and in a depressed patient, that moving forward the sleep-wake cycle had an antidepressant effect (42). Another study investigated the effects of partial SD (PSD) on motor activity, finding that at baseline, responders moved more than non-responders and that all subjects increased activity following PSD (18). One other study, which used ethological methods to assess activation in a pre-SD interview, found the amount of “looking at the interviewer” to be negatively correlated with response to treatment, while observing a positive correlation between “body and object-touching hand movements” and SD response (43). More recently, a study using repeated SD and light therapy in 39 bipolar patients experiencing depressive episodes observed that responders showed increases in activity during the day, and advance in phase of activity-rest rhythms as well as reduced sleep at night after SD, while in contrast, non-responders showed no such changes (44). Another recent paper on 34 patients with major depression did not observe significant differences in activity related to response on the day before SD; during the night, compared to non-responders, responders had increased activity and rested less, and were more active the day after SD (45).

In a recent study, we examined the clinical and genetic factors associated with SD response in a large SD sample (46). In this naturalistic study, SD was conducted in 78 depressed major mood disorder inpatients; 72% responded to SD. Responders and non-responders differed in mood, but not in symptom severity or chronotype. Response was also found to be associated with being younger during SD treatment, and with higher age at time of lifetime disease onset. Significantly higher polygenic risk scores for depression were found in non-responders than healthy controls, while responders had intermediate risk scores.

In the present exploratory secondary analysis, we examined the relationship between SD response and locomotor activity which had been acquired during SD treatment in the same sample (46). Locomotor activity and measurements of subjective mood had been acquired over the course of 1 day as patients underwent SD therapy. Given the equivocal nature of previous reports of locomotor activity in responders and non-responders during SD, we compared the trajectories of locomotor activity in responders *vs.* non-responders. The relationship between locomotor activity and subjective mood was also examined.

## Methods

### Participants

The cohort studied here has been previously described in (46). As an exploratory secondary analysis, we examined locomotor activity data acquired during SD in the same sample. Seventy-eight mood disorder inpatients (34F; age in years mean ± std. dev. = 43.54 ± 14.80, major depressive disorder, n = 71; BD I, n = 6; and BD II, n = 1) experiencing major depressive episodes (ICD-10) were recruited from consecutive admissions to the depression unit of the Department of Psychiatry and Psychotherapy of the Central Institute of Mental Health (CIMH, Mannheim, Germany). Patients had to have been on stable medication regimens for 5+ days prior to undergoing SD. The study was approved by the CIMH ethics committee (II). After being given an explanation of the study, all participants gave written informed consent.

### Sleep Deprivation

**Figure 1:**
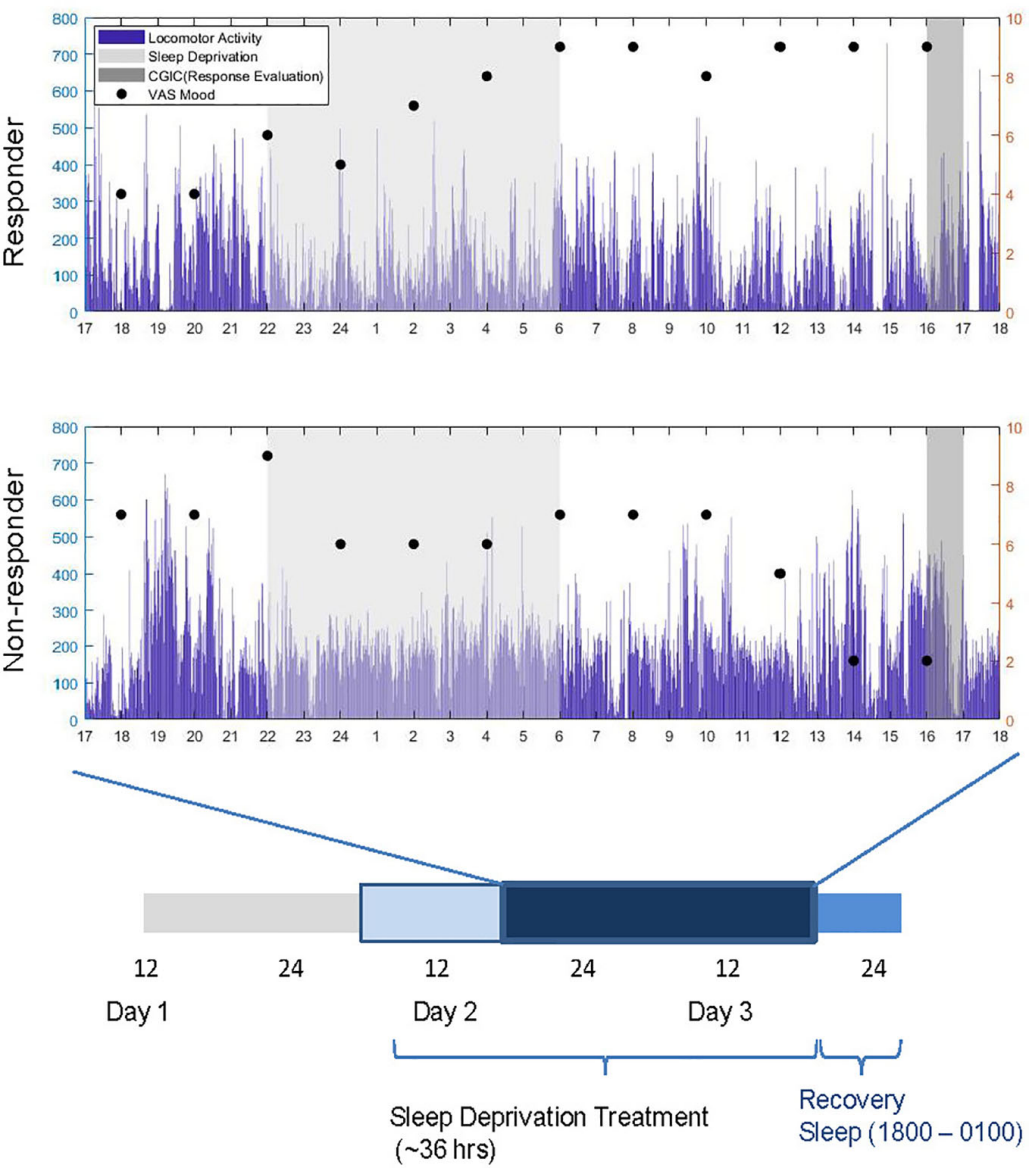
Experimental timeline and sample subjective mood and locomotor data (mg) for a responder and non-responder over the course of sleep deprivation treatment (thicker bar). Response was evaluated between 1600 and 1700 h on Day 3. Dark blue bar denotes time during which locomotor activity was recorded.

Baseline variables were assessed on Day 1 (**Figure 1**). Patients engaged in regular ward activities in the daytime on Day 2. In the evening on Day 2, small groups (1–5 patients) underwent staff-supervised SD. Participants stayed awake for ~36 h from 6:00 am (Day 2) to 6:00 pm (Day 3). On Day 3, patients returned to regular ward activities until they began recovery sleep (6:00 pm–1:00am).

### Data Collection

#### Response to SD

The senior clinical researcher (MD) assessed response to SD using the seven-point Clinical Global Impression Scale of Global Improvement or Change (CGIC) (47) between 4:00 and 5:00pm on Day 3. In the CGIC, the clinician assesses improvement or worsening of the illness relative to before the intervention. Response was dichotomized: response was defined as scores of ≤2 (1: very much improved; 2: much improved) and non-response was define as scores ≥3 (3: minimally improved; 4: No change; 5: Minimally worse; 6: Much worse; 7: very much worse).

#### Locomotor Activity

The SOMNOwatch (SOMNOmedics GmbH, Germany), a wrist-worn actigraph equipped with three activity sensors (x, y, z-axis), from which acceleration data are stored (magnitude, calculated as the square root of x^2^ + y^2^ + z^2^), was used to assess locomotor activity. The range of measurement of the SOMNOwatch is ±8.7 G, and the device has a sensitivity of 0.004 G. Activity was sampled at 60 Hz and stored every 60 s. The devices were worn from 5:00 pm on Day 2 to 6:00 pm on Day 3 (i.e. until recovery sleep, a total of 1,500 min). Patients had been instructed to wear actigraphs at all times except for when they were in the shower or engaging in exercise.

Participants were asked to record if they removed the devices on a wear log. Data were transferred to computer *via* USB connection.

#### Visual Analogue Scale (VAS) Ratings: Subjective Mood

Participants completed a VAS (48) assessing *subjective* mood (hereafter referred to as “mood”) at 2-h intervals from 10:00 am on Day 2 to 6:00 pm on Day 3. Possible ratings ranged from “worst mood imaginable (0)” to “best mood imaginable (10)”; patients indicated their mood in 0.5 point increments. Our previously reported study observed that mood was better in responders than non-responders (46).

### Data Analysis

IBM SPSS Statistics for Windows (v. 25) was used to perform statistical analyses. An alpha of 0.05 was used as the threshold for statistical significance.

Locomotor activity data were preprocessed prior to analysis. Four subjects who were excluded in the previous study for napping were also excluded in the present analysis. No locomotor data was available for five individuals, whose data were excluded from the analysis. Individual data traces were visually inspected. Furthermore, data from patients (n = 7) who had more than 60 consecutive minutes of no activity detected during the recording period were excluded (potentially indicating that they had removed the devices, and perhaps had forgotten to wear them again for a period of time). It was decided that 60 min would be used as a reasonable allowance of time for a patient to engage in exercise or taking a shower.

Mean locomotor activity was calculated for a window of 60 min around (i.e. +/−30 min) VAS mood assessments, as in (31), hereafter referred to as “locomotor activity.” A total of 12 mood assessments (every 2 h from Day 2 at 6:00 pm to Day 3 at 4:00 pm) from each subject were included to match the window recorded locomotor activity. Three subjects for whom no mood data were available were excluded from analysis, giving 59 patients who entered the final analysis, comprising 691 matched mood (a few participants did not indicate mood for several time points) and locomotor activity values. It should be noted that while the total sample was the same as in (46), there were differences in the data available, for example, in the present analysis, patients without locomotor data were excluded, but included in the previous study as they had genetic data.

To explore relationships between response to SD, locomotor activity, and mood, linear mixed models were employed (49). Models with random intercepts and random or fixed slopes for within-subject variables were estimated using maximum likelihood estimation, using a stepwise approach, and the goodness-of-fit of models was compared by using the deviance test on -2 log likelihood. Akaike’s Information Criterion (AIC) was also examined and agreed with the results of the deviance test. Non-significant random effects were not included in the model, and where models did not differ significantly, the more parsimonious model (with fewer effects) was chosen. The resulting main model was specified as follows. Locomotor activity was specified as the dependent variable. *Response* (response/non-response) and *sex* (female/male) were included as fixed factors. *BMI*, *age*, *mood centered around subject mean*, and *mean subject mood* were included as fixed continuous variables. *Time point* and *time point^2^* (to model a circadian effect) were also included as fixed continuous variables. *Time point* was also added as a random effect. Interactions between *Response* x *time point* as well as *response* x *time point^2^* were specified as fixed effects. *Time point* variables were centered to midnight. The intercept was included in the model as a random effect.

To further examine associations of locomotor activity with response to treatment and subjective mood at specific time points, another model was specified with the same fixed factors and continuous variables as the above main model, but with *time point* included as a fixed categorical factor. *Time point^2^* was not included.

## Results

Sixty-nine point five percent of patients (41/59) were evaluated to be responders while 30.5% (18/59) were non-responders. The main mixed model for mean locomotor activity (see **Table 1**) revealed main effects of *response*, with non-responders moving more than responders (t(66.856) = 2.570, p = 0.012). A significant main effect of *time point*^2^ was observed, indicating a U-shaped curve of activity (t(582.872) = 2.730, p = 0.007). A significant main effect of *age* was observed, with increased age associated with decreased locomotor activity (t(58.621) = −2.874, p = 0.006). No significant effects of *subject mean*-c*entered mood*, *mean subject mood*, *sex*, *BMI, and time point* were observed (all p > 0.05). A significant random effect of time point was observed (p = 0.02), revealing variability between subjects in the within-subject relationship between *time point* and locomotor activity. To examine whether diagnosis of bipolar disorder influenced results, the analysis was repeated with a variable differentiating between unipolar/bipolar depression—no significant differences were observed and the variable was not included in the analysis.

**Table 1:** Estimates of fixed effects associated with mean locomotor activity in main model.

Fixed Effects					
Parameter	Estimate	Sth. Error	df	t	Sig.
Intercept	179.927	27.4765	59.003	6.549	**.000**
Non-Responder	29.099	11.323	66.856	2.570	**.012**
Responder ^a^					
Female	-6.508	9.609	58.745	-.677	.501
Male ^a^					
Age	-1.014	.353	58.621	-2.874	**.006**
Body Mass Index	-.474	.796	58.655	-.595	.554
Mean Subject Mood	4.311	3.038	58.728	1.419	.161
Centered Subject Mood	-1.079	1.451	591.884	-.744	.457
Time Point	-.792	1.487	213.188	-.532	.595
Time Point^2^	.590	.216	582.872	2.730	**.007**
Non-response * Time Point	-.133	2.686	212.016	-.049	.961
Response* Time Point ^a^					
Non-response * Time Point^2^	-.468	.396	593.034	-1.183	.237
Response * Time Point^2 a^					
**Random Effects**					
**Parameter**		**Estimate**	**Std. Error**	**Wald Z**	**Sig**.
Residual		2469.881	145.813	16.939	**.000**
Intercept [subject = ID] Variance		1024.446	240.514	4.259	**.000**
Time Point [subject = ID] Variance		25.803	8.412	3.068	**.002**

Mean locomotor activity (mg) 60 minutes around mood assessments (+/- 30 mins) was specified as the dependent variable. Significant (p < 0.05) associations are indicated in bold.

^a^Reference.

**Figure 2:**
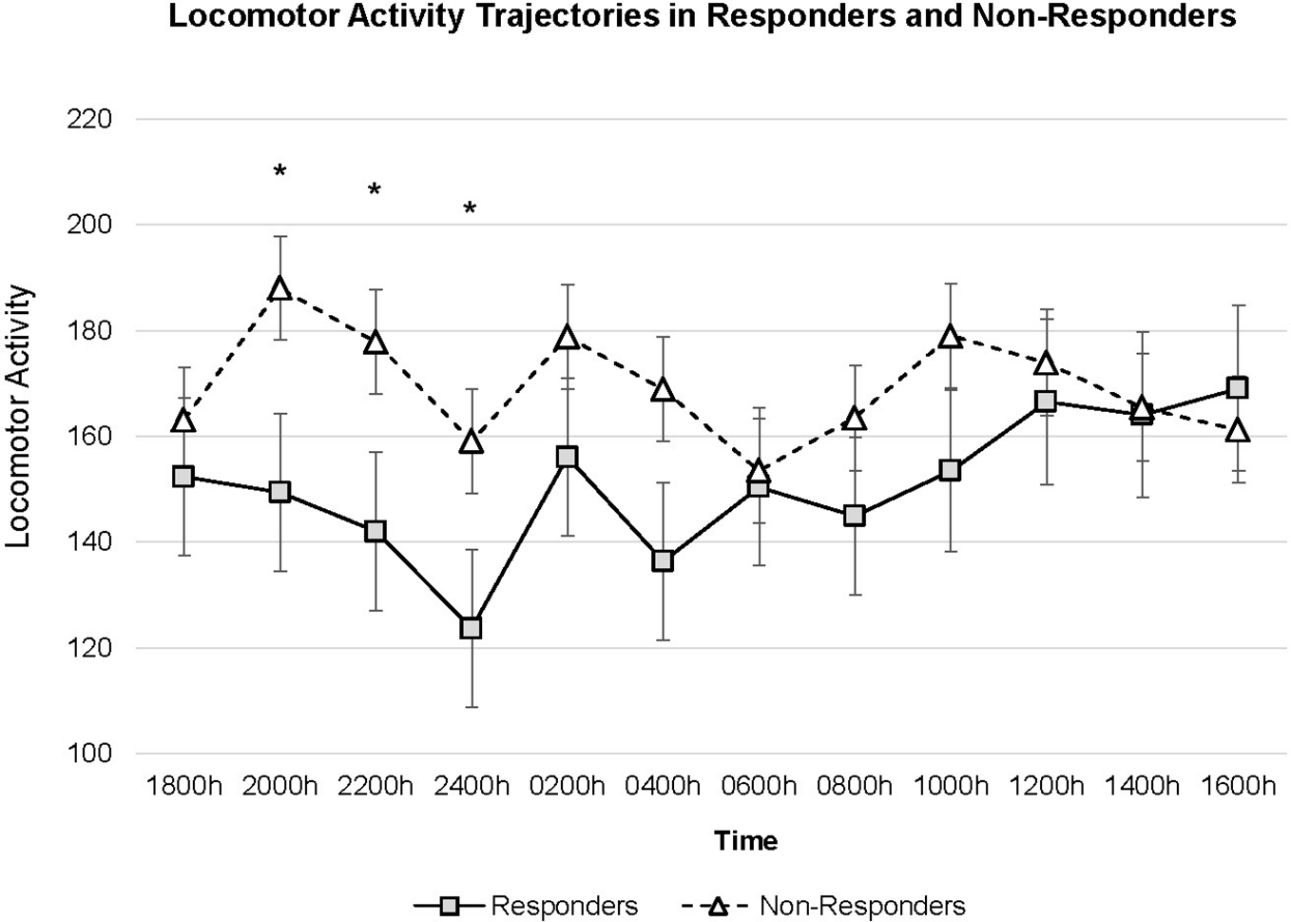
Locomotor activity trajectories for Responders (n = 41) and Non-Responders (n = 18). Figure is drawn based on estimated marginal means calculated in mixed model with time specified as a discrete variable. Error bars denote standard error of mean. ^*^p < 0.05.

The model calculated using *time point* specified as a discrete variable observed only a significant main effect of age (t(58.222) = −2.750, p = 0.008); no other fixed effects were significant. The interaction term between *response* x *time point* revealed higher locomotor activity levels in non-responders the evening of SD (i.e. 2000 h t(632.876) = 2.178, p = 0.030; 2200 h t(632.873) = 2.046, p = 0.041; 2400 h t(632.871) = 2.028, p = 0.043). **Figure 2** shows trajectories of locomotor activity for responders and non-responders.

Next, a *post-hoc* analysis was used to examine whether locomotor activity and mood might be differentially correlated between responders and non-responders. The same model as above was used, stratifying by response status. Mood trajectory (*subject centered mood*) was not significantly associated with locomotor activity in responders (t(440.277) = −1.057, p = 0.291) or non-responders (t(190.845) = 0.882, p = 0.379).

In responders, a significant main effect of *time point^2^* (t(440,710) = 2.559, p = 0.011) and no significant effects of *mean subject mood*, *sex*, *time point*, *age*, and *BMI* were observed. In non-responders, a significant main effect of *age* (t(17.637) = −2.885, p = 0.010) and no significant effects of *time point*, *time point^2^*, *mean subject mood*, *sex*, and *BMI* (all p > 0.05) were observed.

## Discussion

The present analysis examined locomotor activity throughout a 24-h time period as depressed patients underwent SD. We observed higher activity in non-responders compared to responders throughout SD and the differences were most pronounced during the evening of the day of SD until midnight. Activity remained constantly high in non-responders while in responders a dip in activity was observed, followed by an increase over time; the *post-hoc* test stratified for response showed a significant U-shaped curve in responders but not non-responders.

One interpretation of the present observations is that non-responders have more severely disrupted rest-activity cycles. Lower relative amplitude, or difference between activity in active and rest periods, as assessed via actigraphy, has been associated with higher risk for mental health issues and poorer subjective wellbeing (20). It has also been observed that compared to healthy controls, depressed patients have lower activity levels during the day time but higher activity levels at night (23). It is possible that the circadian activity of responders is closer to that of healthy controls, while non-responders may have more disrupted circadian rhythms, thus showing decreased amplitude. Indeed, our findings are in line with a study observing that responders showed changes to activity-rest patterns while non-responders did not (44); blunting of circadian amplitude is suggested to be the main chronobiological abnormality in depression (50).

Unfortunately, many prior SD studies are not directly comparable, as they have used e.g., differing SD regimens (18, 44), actigraphy assessments (see below) and/or different definitions of response (compared to ours and each other). The present findings offer a different perspective from (18), who observed that responders showed higher locomotor activity pre-SD, however, they reported activity during the day and did not record in the late evening, as well as from (45), who reported that responders have higher activity than non-responders during SD. While some studies define response as % reduction in composite scores of ratings such as HAM-D (44, 45) the CGIC (47) performed by experienced clinicians which is a comparative rating well suited to assessing change was used in the present study. As an analysis of pre-existing data, the present study examined locomotor activity only during the SD regimen, in the hospital environment, as opposed to during regular activity. Although differences were observed in the evening before regular sleep hours (i.e. still part of the patient’s “regular” circadian pattern), further investigation outside of the SD protocol will be needed to clarify movement patterns and their meaning.

Studies employing actigraphy in studying response to pharmacological antidepressant treatments (largely using pre-post designs) are also not yet conclusive as to their relationships (51), with some studies observing no changes in motor activity associated with treatment response (52) while others suggest that, for example, the change in quantity of actigraphic movements reflects change in depression psychopathology (53). That said, our results might fit with a previous actigraphic pharmacological study on treatment response, which reported that activity at treatment onset and clinical improvement were negatively correlated, and which observed a trend towards higher activity in nonresponders than responders, and which suggested further exploration of this difference as a predictor of treatment response (54).

It has been proposed that one use of actigraphy might be to identify agitation in depression (23) and the present sample offers an additional point to examine. In our previous report, depressive symptom severity was assessed by clinician (Montgomery-Åsberg Depression Rating Scale) (55) and patient (Beck Depression Inventory-II, BDI-II, self-report version) (56), with consistent significant correlations observed between the scales (46); no differences were observed at baseline between responders and non-responders. The BDI-II contains an item assessing the symptom “agitation.” We conducted an additional post-hoc test (one-way analysis of variance) to examine whether responders and non-responders differed in BDI-II “agitation” scores, finding that baseline (p = 0.698) and post-SD (p = 0.354) scores did not differ between groups. This suggests that results did not reflect differences in agitation (i.e. agitated depression), but it may also be that this is a demonstration of an advantage that actigraphy, as an objective measure, can add to classical assessment methods.

Mood is often positively correlated to locomotor activity in the literature, but this was not found in the present results; this may be explained by several factors. As shown in our previous work (46), subjective mood assessments do not necessarily reflect the same changes as did the global clinical judgement of response. Next, the present mood data were acquired in the midst of the fatigue-inducing SD intervention whereas positive mood-locomotor correlations are often observed in data acquired during daily life. Furthermore, it is uncertain whether mood improvements resulting from SD, changes in locomotor activity, and their relationship, are linear processes with respect both to magnitude and timing and it may also be that the present dataset is too sparse to exhibit associations. Additional research examining different dimensions of mood and assessing specific factors (e.g. depressive symptoms) may help to clarify relationships with locomotor activity.

Varying protocols and devices used so far make it difficult to assess the interaction between locomotor activity and therapeutic responsiveness (57), and this is a relevant factor in interpreting the present findings in context to the literature. A detailed description of different actigraphic methodologies is beyond the scope of this article, and more detail can be found elsewhere (58–60) (**Table S1** provides an overview of actigraphic models used in relevant studies).

Briefly, the digitally integrated magnitude vector acquired in the present study is the most recently developed and yields a more comprehensive measure of physical acceleration than other methods (time-over threshold, zero-crossing), taking into account the amplitude of movement. It is not that one method is necessarily better than the others, but researchers should be aware of the differences between devices as different types and settings can produce very different amounts of activity when recording the same thing (60).

There were several limitations to this study. First, given that this was a naturalistic study and recruitment was done from consecutive admissions, patients were not randomized or stratified during recruitment. Second, the present analysis explored a pre-existing dataset in which the recording period was restricted, providing a useful but limited picture; future research should monitor locomotor activity for extended periods preceding (potentially informing patient screening) and following SD (to examine effects of SD on rest-activity patterns), allowing comparison with baseline and post treatment activity. To better test the utility of locomotor activity as a distinguishing feature which can predict response and non-response, recording during a run-in baseline period will be required. Next, while individual locomotor activity traces were inspected carefully, it may not be possible to definitively distinguish periods of time where participants may have been briefly resting, from when they may have taken short naps, using actigraphy alone. This issue requires further examination. It has been observed that naps potentially disrupt a successful SD response and can reverse antidepressant effects (61). While this was not an issue during night-time supervised SD and during ward activities the next day, we cannot exclude that brief naps may have occurred in some of the patients, possibly leading to early relapse to depression and thus potential misclassification of responders as non-responders. However, given clinical protocols, we believe that misclassification is unlikely—patients were seen in ward rounds the morning after SD and given CGI ratings in the afternoon. The CGI was assessed by a clinician very familiar with the patient and also based on reports about SD response from staff on the ward accompanying the patient throughout the night.

Prior findings and those from the present study suggest to further explore the correlation between locomotor activity and mood and its potential role as a predictor of treatment response. Availability and use of wearable and mobile sensing devices has increased exponentially (38), and given the suitability of the devices and the ambulatory assessment approach (62, 63) to longitudinally study sleep and circadian disruptions, they should be employed further to study psychopharmacologic effects (57). We propose that application of such an approach, augmented with the acquisition of additional physiological, biological, and behavioral factors will go beyond the identification of predictors and eventually lead to better understanding of mechanisms behind not only fast-acting treatments such as SD, but of disease mechanisms on a larger scale.

## Data Availability Statement

The data analyzed in this study is subject to the following licenses/restrictions: privacy regulations. Requests to access these datasets should be directed to the corresponding author.

## Ethics Statement

The studies involving human participants were reviewed and approved by Central Institute of Mental Health Ethics Committee (II). The patients/participants provided their written informed consent to participate in this study.

## Author Contributions

JF: formal analysis, writing-original draft, review and editing (R&E), visualization. LS: writing-R&E. NT: investigation, writing-R&E. JK: formal analysis, writing-R&E. SW: conceptualization, methodology, writing-R&E. FS: writing-R&E. JF: writing-R&E. LZ: writing-R&E. AM-L: resources, writing-R&E. UE-P: writing-R&E. CS: resources, writing-R&E. MS: resources, writing-R&E. YY: writing-R&E. MG: project administration, supervision, investigation. MD: writing-R&E, project administration, supervision, investigation, conceptualization, methodology, funding acquisition. MR: writing-R&E, supervision, investigation, conceptualization, methodology, funding acquisition.

## Funding

This study was supported by the German Federal Ministry of Education and Research (BMBF) under the e:Med Programme (Target-OXY: 031L0190A) and ERA-NET NEURON (EMBED: 01EW1904 and SYNSCHIZ: 01EW1810). AM-L acknowledges support from the BMBF (Grants 01ZX1314G, 01GS08147, 01EF1803A, 255156212 CRC 1158 subproject B09) and the Ministry of Science, Research and the Arts of the State of Baden-Wuerttemberg, Germany (MWK, grants 42-04HV.MED(16)/16/1, 42-04HV.MED(16)/27/1). The funders had no involvement in the study design, collection or analysis and interpretation of data, writing of the report, or decision to submit the manuscript for publication.

## Conflict of Interest

AM-L has received consultant fees from Boehringer Ingelheim, Brainsway, Elsevier, Lundbeck Int. Neuroscience Foundation and Science Advances.

The remaining authors declare that the research was conducted in the absence of any commercial or financial relationships that could be construed as a potential conflict of interest.
