# Supplementary material for: Association of Locomotor Activity During Sleep Deprivation Treatment With Response
Source: Front Psychiatry. 2020 Jul 21;11:688. doi: 10.3389/fpsyt.2020.00688 (PMC7385277; doi:10.3389/fpsyt.2020.00688)
Supplement: Supplementary file 1 [file Table_1.docx]

| **Table S1. Relevant Studies Assessing Locomotor Activity in Depressed Populations** | | | | |
| --- | --- | --- | --- | --- |
| **Author** | **Title** | **Year** | **Sample size** | **Used Recording device and settings (if specified)** |
| Baune BT, Caliskan S, Todder D | Effects of adjunctive antidepressant therapy with quetiapine on clinical outcome, quality of sleep and daytime motor activity in patients with treatment-resistant depression | 2007 | N= 27 with major depressive disorder | - Actigraph (Actiwatch-S, Cambridge Neurotechnology Ltd, Cambridge, UK) - Signal detecting every 30 seconds |
| Benedetti F, Dallaspezia S, Fulgosi MC, Barbini B, Colombo C, Smeraldi E | Phase advance is an actimetric correlate of antidepressant response to sleep deprivation and light therapy in bipolar depression | 2007 | N= 39 inpatients affected by bipolar disorder type I | - Activity monitors (Mini Motionlogger Actigraphs; Ambulatory Monitoring, Inc., Ardsley, New York, USA) - Data collection in Zero-Crossing Mode in 1 min epochs |
| Berle JO, Hauge ER, Oedegaard KJ, Holsten F, Fasmer OB | Actigraphic registration of motor activity reveals a more structured behavioural pattern in schizophrenia than in  major depression | 2010 | N= 23 schizophrenia patients,  N=23 major depressive episode,  N= 32 controls | - Actiwatch, Cambridge Neurotechnology Ltd, England - Activity counts were recorded for one minute intervals |
| Brückner TU, Wiegand MH | Motor activity in depressed patients during therapeutic sleep deprivation | 2010 | N=34 major depression inpatients | - Actigraph (3DbioLOG by Gefatec) - Sampling every 2 minutes |
| Gershon A, Ram N, Johnson SL, Harvey AG, Zeitzer JM | Daily Actigraphy Profiles Distinguish Depressive and Interepisode States in Bipolar Disorder | 2016 | N= 37 bipolar disorder type I,  N= 39 no lifetime mood disorder | - Actiwatches (AW64, Respironics Inc., Bend OR) - Piezoelectric sensor - Activity count over one minute intervals |
| Indic P, Murray G, Maggini C, et al. | Multi-scale motility amplitude associated with suicidal thoughts in major depression | 2012 | N= 36 depressed subjects | - Actigraphic device (AMI-128K from Mini-MotionloggerH Actigraph, Ambulatory Monitoring, Inc. [AMI], Ardsley, NY,USA) - Piezoelectric signals, sampled at 32 Hz in 6-minute epochs |
| Kim J, Nakamura T, Kikuchi H, Sasaki T, Yamamoto Y | Co-variation of depressive mood and locomotor dynamics evaluated by ecological momentary assessment in healthy  Humans | 2013 | N= 113 healthy subjects | - Watch-type computer (Ruputer, ECOLOG, 42 g; Seiko Instruments Inc., Tokyo, Japan), equipped with an activity monitor (Ambulatory Monitors Inc., Ardsley, NY, USA) - Uni-axial piezoelectric accelerometer - Zerocrossing counts accumulated in 1-min epoch |
| Kim J, Nakamura T, Kikuchi H, Yoshiuchi K, Sasaki T, Yamamoto Y | Covariation of depressive mood and spontaneous physical activity in major depressive disorder: toward  continuous monitoring of depressive mood | 2015 | N = 14 patients with major depressive disorder,  N= 43 healthy subjects | - A watch-type device, where also mood assessments can be indicated was used to capture activity (Ambulatory Monitors Inc., Ardsley, NY, USA) - Activity is assessed uniaxial, zero-crossing counts for every minute were summed. |
| Krane-Gartiser K, Henriksen TE, Vaaler AE, Fasmer OB, Morken G. | Actigraphically assessed activity in unipolar depression: a comparison of inpatients with and without motor retardation | 2015 | N= 52 admitted inpatients with unipolar depression,  N= 28 healthy controls | - Actigraph (Actiwatch Spectrum, Philips Respironics Inc, Murrysville Pennsylvania) - Piezoelectric accelerometer - Activity counts for one minute intervals |
| Raoux N, Benoit O, Dantchev N, et al. | Circadian pattern of motor activity in major depressed patients undergoing antidepressant therapy: relationship between actigraphic measures and clinical course | 1994 | N= 26 with major depression | - Motor activity monitor - Movement signals are integrated over usually 7.5 minutes - As described in Borbely et al.,1981 |
| Razavi N, Horn H, Koschorke P, Hügli S, Höfle O, Müller T, Strik W, Walther S | Measuring motor activity in major depression: The association between the Hamilton Depression Rating Scale and actigraphy | 2011 | N= 76 medicated inpatients with major depression | - An actigraph (Actiwatch®, Type AW4, Cambridge Neurotechnology Inc., UK) - Integrated accelerometer produced voltage whenever the actigraph is moved |
| Reichert M, Lutz A, Deuschle M, et al. | Improving motor activity assessment in depression: which sensor placement, analytic strategy and diurnal time frame are most  powerful in distinguishing patients from controls and monitoring treatment effects | 2015 | N= 27 depressed patients, N= 16 healthy controls | - Portable physiological recorder-analyser system, the Varioport-B (Becker Engineering, Karlsruhe, Germany) - Sampling at 32 Hz |
| Szuba MP, Baxter LR, Fairbanks LA, Guze BH, Schwartz JM | Effects of partial sleep deprivation on the diurnal variation of mood and motor activity in major depression | 1991 | N= 24 unipolar depression, N= 9 bipolar I depression, N= 4 bipolar II depression | - Data storage via portable microcomputer in a belt-worn pack, attached to the motion sensor (Vitalog Corporation, Redwood City, CA) - Omnidirectional mercury tilt switch |
| Teicher MH, Glod CA, Magnus E, Harper D, Benson G, Krueger K, et al. | Circadian rest-activity disturbances in seasonal affective disorder | 1997 | N= 20 healthy controls, N= 25 outpatients meeting criteria for seasonal affective disorder and major or bipolar depression with seasonal pattern | - Activity monitoring using the Colburn-National Institute of Mental Health Piezoelectric monitor, MIT, Cambridge - Activity counts were accumulated over 15 minute periods |
| Todder D, Caliskan S, Baune BT | Longitudinal changes of day-time and night-time gross motor activity in clinical responders and non-responders of major depression | 2009 | N= 27 patients with depression, N= 27 healthy controls | - Actiwatch- S (Cambridge Neurotechnology Ltd, Cambridge, UK) - Signal detection every 30s |
| Volkers AC, Tulen JHM, van der Broek WW, Bruijn JA, Passchier J, Pepplinkhuizen L | Motor activity and autonomic cardiac functioning in major depressive disorder | 2003 | N= 67 unmedicated unipolar depressed inpatients, N= 64 control subjects | - Actigraph (Gaehwiler Electronic) - Integrated monoaxial piezoelectric acceleration sensor |
| Walther S, Hügli S, Höfle O, et al. | Frontal white matter integrity is related to psychomotor retardation in major depression | 2012 | N= 21 medicated patients with major depressive disorder, N= 21 matched controls | - Actigraph (Actiwatch®, Cambridge Neurotechnology, Inc., UK) - Activity counts stored in 2 seconds intervals |
